# The Acceptability of Occupational Therapy Using Zones of Regulation™ Concepts in an Equine Environment to Autistic Children

**DOI:** 10.3390/bs15040495

**Published:** 2025-04-08

**Authors:** Jacqueline M. Browne, Sarah Jean Reega, Ellen M. Rankins, Arlene A. Schmid, B. Caitlin Peters

**Affiliations:** 1 Department of Occupational Therapy, Colorado State University, Fort Collins, CO 80523, USAarlene.schmid@colostate.edu (A.A.S.); 2 Temple Grandin Equine Center, Department of Animal Sciences, Colorado State University, Fort Collins, CO 80523, USA; sarah.reega@colostate.edu (S.J.R.); ellen.rankins@colostate.edu (E.M.R.); 3 Colorado State University Spur Campus, Denver, CO 80216, USA

**Keywords:** occupational therapy, equine-assisted services, autism, acceptability, self-regulation

## Abstract

Autistic children often have impaired self-regulation which can impact daily functioning and life outcomes. Occupational Therapy Using Zones of Regulation™ Concepts in an Equine Environment (OT-ZOR-Equine) is a standardized intervention that integrates both the Zones of Regulation™ curriculum and horses into occupational therapy to address self-regulation in autistic children. We aimed to assess the acceptability of OT-ZOR-Equine to participating autistic children. A qualitative descriptive research study was conducted with six autistic children ages 7–9 years who received ten weeks of OT-ZOR-Equine. Children participated in semi-structured interviews that used questions guided by the Theoretical Framework of Acceptability. We analyzed interview transcripts using directed content analysis. Autistic children found OT-ZOR-Equine to be generally acceptable. The children especially enjoyed horse riding but found the Zones of Regulation™ curriculum and certain aspects of riding to be less acceptable. Tailoring OT-ZOR-Equine to integrate each client’s preferences and needs may make this intervention more acceptable to autistic children. The findings of this study support the continued use of and research on OT-ZOR-Equine or similar occupational therapy interventions that integrate horses to influence self-regulation in autistic children.

## 1. Introduction

Approximately one out of every thirty-six children in the United States has been identified as having autism (Centers for Disease Control and Prevention, 2023). Individuals with autism may have difficulty with social communication and demonstrate restricted, repetitive behaviors (APA, 2013). Although not part of the diagnostic criteria, impaired self-regulation is increasingly recognized as a prominent aspect of autism (Laurent & Gorman, 2018; Mazefsky & White, 2013; Samson et al., 2014, 2015). Self-regulation refers to the capacity to monitor, assess, and alter one’s emotional state, arousal level, and actions in order to perform goal-directed behaviors (Laurent & Gorman, 2018; Hofmann et al., 2012). For individuals with autism [referred to as “autistic” moving forward (Bottema-Beutel et al., 2021), impaired self-regulation is often exhibited in increased physiological responsiveness to everyday activities (Corbett et al., 2006; Spratt et al., 2012) and challenges with emotional management (Beck et al., 2020; Cai et al., 2018). Difficulty with self-regulation also presents in autistic children as behavioral differences, such as elevated hyperactivity, agitation, elopement, combativeness, or self-harm (Doehring et al., 2014; Mazefsky & White, 2013; Mazefsky et al., 2014). Difficulty with self-regulation can influence autistic children’s daily functioning and overall well-being, as impaired self-regulation is linked to lower social adjustment (Nader-Grosbois & Mazzone, 2014), diminished academic performance (Konstantareas & Stewart, 2006), and higher levels of anxiety (Swain et al., 2015). Compared to non-autistic individuals, autistic children also have increased difficulty implementing self-regulation strategies to handle everyday encounters (Jahromi et al., 2012; Samson et al., 2014, 2015). Thus, it is important to utilize therapeutic interventions that support autistic children’s development and use of adaptive self-regulation strategies (Samson et al., 2015).

Occupational therapists support self-regulation in autistic children by helping them understand and learn strategies to modify their emotional state and degree of physiological arousal (Martini et al., 2016). One increasingly popular occupational therapy intervention used to address self-regulation in this population is occupational therapy in an equine environment (OTEE) (Lindley et al., 2018). During OTEE, occupational therapists use the movement of a horse and related activities (e.g., grooming, saddling, etc.) as tools to address functional outcomes (McDaniel Peters & Wood, 2017; Ajzenman et al., 2013). There are promising preliminary outcomes of OTEE for autistic children, including increased adaptive behaviors (Ajzenman et al., 2013), increased social motivation (Peters et al., 2022), improved occupational performance in individual goal areas (Peters et al., 2022), improved task engagement (Llambias et al., 2016), increased participation in everyday activities (Ajzenman et al., 2013), and improved posture (Ajzenman et al., 2013). Despite these promising outcomes, the existing literature on OTEE largely encompasses pilot studies without active comparison groups or standardized intervention protocols. To address these gaps in research and to specifically study the efficacy of OTEE for improving self-regulation in autistic children, our research team developed Occupational Therapy using Zones of Regulation™ Concepts in an Equine Environment (OT-ZOR-Equine; Peters et al., 2024b). OT-ZOR-Equine is a standardized intervention protocol that integrates “(1) evidence-based practices relevant to occupational therapy for autistic children, (2) concepts from the Zones of Regulation™ curriculum, and (3) purposeful integration of horses into occupational therapy” (Peters et al., 2024b, p. 3). The Zones of Regulation™ curriculum helps children to identify emotions, group emotions within four specific “zones”, and use strategies (“tools”) for emotional management (Kuypers, 2011; Peters et al., 2024b).

In a preliminary efficacy study, OT-ZOR-Equine was found to improve self-regulation and social functioning in autistic children (Peters et al., 2024b). An ongoing randomized controlled trial (RCT; NCT05846932) is further studying the efficacy of OT-ZOR-Equine. However, the existing literature and the ongoing study (NCT05846932) have not included the perspectives of the autistic children who participate in OT-ZOR-Equine. Rather, parent-report has been utilized to measure outcomes and intervention acceptability (Peters et al., 2024b). Specifically, a previous study of 14 autistic youth and their parents found that parents were satisfied with OT-ZOR Equine, expressing that it was a good fit for their child, their child enjoyed it, it was beneficial, and that they would recommend the intervention to other caregivers of autistic youth. Further, there was a 100% participant retention rate and 95% attendance rate, demonstrating good adherence to the intervention. However, the acceptability of OT-ZOR Equine from the perspective of the children who actually participate in the intervention has not been studied. There is increasing recognition of the importance of including autistic voices in autism intervention research (Chown et al., 2017), as exemplified by the recent “Evidence, Ethics, and Effectiveness of Autism Interventions” Executive Summary that states “intervention research should address the autistic person’s experience of the intervention, including their internal experiences and wellbeing” (Autistic Self Advocacy Network, 2022). Therefore, it is necessary to understand the acceptability of OT-ZOR-Equine from the perspective of autistic children.

Intervention acceptability refers to the “extent to which people delivering or receiving a healthcare intervention consider it to be appropriate, based on anticipated or experiential cognitive and emotional responses to the intervention” (Sekhon et al., 2017, p. 5). It is imperative to assess the acceptability of a healthcare intervention to those participating in the intervention, as acceptability may influence execution, adherence, results, and efficacy of an intervention (Sidani & Braden, 2011; Sekhon et al., 2022). Sekhon et al. (2017) developed the Theoretical Framework of Acceptability to operationalize the acceptability of healthcare interventions. In the framework, acceptability is composed of seven constructs: (1) affective attitude, (2) burden, (3) ethicality, (4) perceived effectiveness, (5) intervention coherence, (6) self-efficacy, and (7) opportunity costs.

The purpose of this study was to examine the acceptability of OT-ZOR-Equine from the perspective of autistic children who participated in the intervention. To accomplish this purpose, we selected four of the constructs from Sekhon et al.’s (2017, 2022) Theoretical Framework of Acceptability that were the most concrete, and therefore easiest for autistic children to understand. Affective attitude refers to “how an individual feels about the intervention” (Sekhon et al., 2022, p. 5); burden refers to “the amount of effort required to participate in the intervention” (p. 5); perceived effectiveness refers to “the extent to which the intervention has achieved its intended purpose” (p. 5); and self-efficacy refers to “the participant’s confidence that they can perform behaviors required to participate in the intervention” (p. 5). Therefore, to assess these four aspects of intervention acceptability, we asked the following research questions:

1. What are the participants’ attitudes towards OT-ZOR-Equine?
2. How much effort was required for children to participate in OT-ZOR-Equine?
3. To what extent did participants perceive OT-ZOR-Equine to influence their self-regulation?
4. How confident, or unconfident, did participants feel in their ability to participate in OT-ZOR-Equine?

## 2. Materials and Methods

We implemented a qualitative descriptive research approach. This approach is effective when researchers aim to provide straightforward and accurate descriptions of participants’ experiences or perspectives of a phenomenon, especially a minimally studied phenomenon (Sandelowski, 2000, 2010; Kim et al., 2016). To collect data, the qualitative descriptive research method frequently uses semi-structured interviews in which open-ended questions are guided by a specific framework (Sandelowski, 2010). In this study, interview questions were based on a Theoretical Framework of Acceptability put forth by Sekhon et al. (2017, 2022). Consistent with a qualitative descriptive approach, data analysis consisted of a qualitative content analysis. In this method, investigators analyze the data using subtle interpretations, to stay as near to the participants’ original words as possible (Sandelowski, 2000; Kim et al., 2016). These “data-near interpretations” separate qualitative content analysis apart from approaches that utilize theory-driven or more complex levels of interpretation such as grounded theory or phenomenology (Sandelowski, 2010, p. 79).

The Institutional Review Board of Colorado State University approved the procedures of this qualitative descriptive study.

### 2.1. Participants

We recruited autistic children from an ongoing RCT (NCT05846932). As part of their participation in the RCT, all children had previously been screened to ensure they met the following criteria: ages 6–11, diagnosed with autism, met clinic cut-offs for autism on the Autism Diagnostic Observation Schedule (ADOS) and Social Communication Questionnaire, had a non-verbal IQ ≥ 65, were verbally fluent as defined by the ADOS, experienced irritability, and had not ridden horses for 10+ hours in the past 6 months. Once children in the Summer 2024 cohort completed at least eight weeks of OT-ZOR-Equine (*n* = 6), they were eligible to complete the current study, distinct from the RCT, which consisted of a qualitative interview. Parents received an IRB-approved informational flyer about this study during week eight of the 10-week intervention. Interested participants verbally informed ER they were interested in the study during week eight or nine of the OT-ZOR-Equine intervention, or they followed up via phone call or email as listed on the flyer. ER scheduled participants for an in-person or online interview, depending on participant preference.

One week prior to the interviews, JB emailed parents consent and assent forms as well as the open-ended interview questions to review beforehand. Just prior to the interviews, parents (five mothers and one father) consented to have their child participate in interviews, and all six children provided either verbal or non-verbal assent. Upon completion of the interview, participants received a $20 gift card and merchandise (hat, stuffed animal, or mug) from Temple Grandin Equine Center (TGEC). Table 1 provides demographic and clinical information of the children who enrolled in the study.

### 2.2. The Intervention

The OT-ZOR-Equine intervention was first described by our team in Peters et al. (2024b) and is summarized here. The intervention was delivered to participants by an occupational therapist with training from the American Hippotherapy Association, Inc. for an hour, once a week for ten weeks during an ongoing RCT (NCT05846932). Each session included the following: (1) a caregiver check-in while the child provided a pre-test saliva sample, (2) 45 min of therapeutic activities mounted on a horse, and (3) caregiver education while the child provided a post-test saliva sample. The mounted therapeutic activities in all sessions were guided by the following critical elements:

1. Use of equine movement and the participant’s interests (including preferred equine activities) to optimize attention and engagement in the therapy session.
2. Direct instruction of a self-regulation skill (see list below).
3. Therapeutic activities that integrate horses to practice the week’s targeted self-regulation skill (minimum of 40 min mounted; trotting optional but often included; minimum of three opportunities to practice the skill).
4. Individualized positive reinforcement for practicing the self-regulation skill.
5. Scaffolding skill performance using prompting, fading, shaping, chaining, and feedback.
6. Arrangement of the environment to best support skill performance (i.e., horse selection, tack selection, arena set-up).

Each week, the therapy session focused on a distinct self-regulation skill, drawn from the Zones of Regulation curriculum (Kuypers, 2011), including:

1. Categorizing emotions and alertness states into four “Zones”
2. Understanding the purpose of a regulation “tool”
3. Recognizing emotions in others
4. Identifying common emotional responses to various situations
5. Identifying individualized body cues for different “Zones”
6. Identifying sensory self-regulation tools
7. Identifying calming self-regulation tools
8. Identifying cognitive self-regulation tools
9. Creating an individualized self-regulation toolbox
10. Using an individualized self-regulation toolbox

### 2.3. Data Collection

As part of the enrollment process for the OT-ZOR-Equine RCT (NCT05846932), participants completed a demographic form and several standardized assessments, which were used to characterize their baseline clinical presentation. Parents completed the Adaptive Behavior Assessment System, Third Edition (ABAS-3), an assessment of adaptive skills (Harrison & Oakland, 2015), and the Social Communication Questionnaire (SCQ) which screens for autism spectrum disorder (Rutter & Bailey, 2003). ER administered the Leiter-3 to all participants to characterize their non-verbal IQ (Roid et al., 2013).

For the current study, JB conducted semi-structured interviews with participants within one week of their completion of the 10-week OT-ZOR-Equine intervention. Four participants completed in-person interviews, and two participants completed online interviews via Microsoft Teams™ (version 24180.210.3001.6635). Mothers of five participants and the father of one participant were present for the duration of their child’s interview. Interviews ranged from ten to eighteen minutes, occurred in English, and were audio recorded via Microsoft Teams™. Participants were welcomed to play with toys or eat a snack to increase their comfort throughout the interview. Interviews included open-ended questions that were adapted from the Theoretical Framework of Acceptability (Sekhon et al., 2017, 2022) and were reworded in a semi-structured interview guide (Table 2) to be age-appropriate for autistic children. Thus, we asked participants about (a) their attitude towards OT-ZOR-Equine, (b) their perceived burden to participate in OT-ZOR-Equine, (c) their perceptions on the influence of OT-ZOR-Equine on their self-regulation, and (d) their confidence in participating in OT-ZOR-Equine. When participants only utilized body language (i.e., head nod) to answer a question, JB verbally reflected their response back to them (i.e., “yes?”, “no?”, “I see you shaking your head yes”) and made a fieldnote indicating that the participant utilized non-verbal answers during the interview. Some participants demonstrated difficulty producing answers to various questions throughout the interviews, likely due to impaired social communication common in autistic children. Therefore, for at least one question in each interview, JB verbally provided participants with an array of potential responses (i.e., “easy”, “medium”, or “hard”) they could choose to use and supplied examples of activities completed throughout the intervention to support participants’ recall of the intervention. Three parents naturally provided similar assistance throughout the interview (i.e., without prompting to do so by the research team), reminding their child of the activities they engaged in during OT-ZOR-Equine or prompting them with a set of possible answers. Additionally, an age-appropriate definition of the word “confidence” was provided to participants if they did not know what confidence meant. JB also told participants that they could choose to skip any questions throughout the interview, and that there were no right or wrong answers. The first six questions and responses of one participants’ interview were unintentionally not recorded due to user error of Microsoft Teams’™ recording function; however, her responses to the remaining fifteen questions were recorded.

Transcripts from each interview recording were downloaded from Microsoft Teams™. JB listened to the audio recordings repeatedly while concurrently reading the transcripts to identify and correct any errors in the transcripts. JB de-identified the transcripts by replacing names with a pseudonym. De-identified transcripts were then saved in a Microsoft Word™ document, and uploaded to NVivo version 14, a qualitative data analysis software program.

### 2.4. Data Analysis

Qualitative content analysis is the preferred method of data analysis in qualitative descriptive research (Kim et al., 2016; Sandelowski, 2000). We implemented a directed content analysis, a type of content analysis that utilizes an existing theory or framework to deductively derive codes or categories (Hsieh & Shannon, 2005). Therefore, we created four a priori categorical codes based on the four selected constructs from the Theoretical Framework of Acceptability (Sekhon et al., 2017, 2022): (1) the participant’s attitude towards the intervention, (2) their perceived burden to engage in the intervention, (3) the perceived effectiveness of the intervention, and (4) the participant’s self-efficacy in participating in the intervention. Multiple subcodes under each a priori categorical code were also deductively created based on the Theoretical Framework of Acceptability and defined in NVivo prior to the initial round of coding. During the first round of coding, the interview transcript data were reviewed line-by-line by JB, using the categorical codes and subcodes to group text into categories. Categorical codes and subcodes were also inductively developed from the data during the initial round of coding. After the initial coding round, all codes were peer reviewed with BCP, and codes were combined and redefined into the final coding scheme. JB and SJR then separately reviewed all transcripts to apply the final coding scheme. The coding of JB and SJR were compared to examine intercoder agreement. There was substantial agreement between 85.54% of codes (kappa > 0.6) and excellent agreement between 78.29% of codes (kappa > 0.8). Any discrepancies in coded data were discussed among the three researchers until an agreement was reached. We decided that one new code that arose inductively during SJR’s initial coding process should be part of the final coding scheme. This code related to a participant feeling “silly” during the intervention and was applied to the final coding system. We also came to the consensus that any of the participants’ responses that were unclear or provided conflicting information were not included in the final coding scheme, as we did not want to incorrectly represent what the participant was trying to communicate. Finally, we agreed to code the interviewer’s response to participants’ non-verbal body language (i.e., head nod) when the participant did not provide a verbal response. The codes were then reviewed and compared within and between categories and subcategories to synthesize the findings both narratively and through tables.

### 2.5. Trustworthiness

We used various strategies to strengthen the trustworthiness of this study. We engaged in reflexivity throughout the study, considering our positionality and experience related to autistic children and OT-ZOR-Equine, which may have shaped the interpretation and approach of this study (Creswell & Poth, 2018). JB’s experience supporting autistic children and utilizing the Zones of Regulation™ curriculum in equine environments and her doctoral level education in occupational therapy shaped her approach to interviewing participants and may have influenced data analysis. Likewise, as the creator of OT-ZOR-Equine with experience implementing this intervention with several cohorts of research participants, BCP utilized her expertise to guide this study’s methodology and data analysis. SJR, the secondary coder, was a PhD candidate in Animal Sciences with a focus on equine-assisted services at the time of data analysis. She was less experienced with implementing OT-ZOR-Equine but still knowledgeable about the content of the intervention. Aware of their favorable opinions toward equine-assisted services, JB, SJR, and BCP paid special attention to capture any potentially negative comments about OT-ZOR-Equine during both the interviews and data analysis. Additionally, throughout the data analysis process, JB documented assumptions and interpretations within memos in NVivo. The memos, interview transcripts, and descriptions of codes were all utilized as an audit trail. We conducted peer reviews of interview questions and the coding scheme in weekly research team meetings to check accuracy and to limit the effect of researchers’ positionality on data collection and analysis (Creswell & Poth, 2018; Bhattacharya, 2017).

We also used data triangulation to enhance the validity of the findings. This was achieved through (1) data source triangulation (collecting data from six participants), (2) investigator triangulation (JB and SJR independently analyzing and remaining blinded to each other’s initial coding), and (3) methodological triangulation (comparing findings from this study with findings from previous studies that reported on acceptability of OT-ZOR-Equine from parental perspectives; see Section 4; Creswell & Poth, 2018). Discussing and resolving any discrepancies in JB and SJR’s coding allowed us to reach intercoder agreement which enhanced the reliability of the results (Creswell & Poth, 2018). Integrating exact quotations of autistic children into the results contributed to rich descriptions of the participants’ perspectives and reinforced our data-near interpretations. These dense descriptions strengthen the transferability of this study (Creswell & Poth, 2018).

## 3. Results

All six participants who completed the OT-ZOR-Equine intervention in Summer 2024 (NCT05846932) enrolled and completed the current study. Four primary themes arose from the data. Each theme summarized important concepts connected to the a priori categorical codes used to direct the data analysis process. The first theme focused on the participants’ attitudes towards the intervention. The second theme focused on the children’s perceived effort to participate in the intervention. The third theme focused on participants’ perceptions of how the intervention influenced their self-regulation, and the fourth theme focused on the children’s self-efficacy in participating in the intervention.

### 3.1. Participants’ Attitudes Towards the Intervention

Table 3 provides selected participant responses because they illustrate participants’ attitudes towards the intervention. Overall, the children had a primarily positive attitude towards OT-ZOR-Equine. All six children affirmed or expressed enjoyment of engaging in the intervention. The participants especially liked riding and interacting with the horse, and they mentioned multiple preferred factors, including trotting, the sensory experience of riding or engaging with the horse, the horse’s demeanor, riding backward, and playing games while mounted on the horse (see Table 3). However, four participants also noted non-preferred aspects of riding. In week seven of the 10-week intervention, Ben fell off the horse when the horse spooked to the sound of a falling broom nearby; the horse sidestepped quickly, causing the participant to fall to the ground. Ben did not sustain any injuries, but he expressed during the interview that he was nervous in the session following the fall when his horse “twitch[ed]”. John also felt nervous about the potential of falling off a large horse during the first week of the intervention, but expressed he became more comfortable with time. He also disliked the amount of dirt on the horse, or when he rode a non-familiar horse (due to his usual horse being unavailable one day). Joe felt uncomfortable sitting backward in the saddle during an activity, and Hanna did not like getting off the horse. Ben and Joe stated that there was nothing that they did not enjoy about riding. Despite some non-preferred aspects of riding the horse, all participants affirmed that they would like to return to TGEC to ride a horse again.

All of the participants who responded to the question (n = 5) stated that they enjoyed engaging with the occupational therapists. John explained that his therapist ensured he was safe, and Ben liked the therapist’s instructions throughout the intervention. Maya liked when the therapist put her helmet on, and John and Ben mentioned that their therapist was “nice”.

There were varied attitudes towards the Zones of Regulation™ curriculum that was taught throughout the intervention. Three participants liked learning about the Zones of Regulation™ curriculum. However, only Sam stated that he would like to return to TGEC to learn about the Zones of Regulation™ curriculum again. All other participants indicated that they would not want to engage in Zones of Regulation™ activities again. John and Joe felt that the Zones of Regulation™ curriculum was boring, as John explained that he had previously learned about it at school. Joe also stated that he “hate[d]” learning about the self-regulation skills.

Participants shared diverse suggestions for improving the enjoyment or comfort of the OT-ZOR-Equine intervention. Four participants gave recommendations for the horse-riding experience. John explained that he enjoyed riding but would prefer it if the time spent mounted on the horse was shorter, less messy, and did not include the Zones of Regulation™ activities. Sam suggested “trotting”, and Joe suggested galloping to make riding more fun. Hanna suggested having comfort items (i.e., her stuffed animal or a snack) while on the horse or jumping on the trampoline during the intervention. Maya had no suggested additions for improving the comfort or enjoyment of riding. Furthermore, four participants had no suggestions to make the experience of learning the Zones of Regulation™ curriculum more comfortable or engaging, but two children suggested additions for making the Zones of Regulation™ activities more fun. Joe explained that he would enjoy galloping during the Zones of Regulation™ activities, and Hanna thought it would be more fun to have her stuffed animal with her on the horse.

### 3.2. Burden to Participate in the Intervention

Table 4 provides selected participant responses that illustrate participants’ perceived effort to participate in OT-ZOR-Equine. The burden, or perceived amount of effort required to participate in OT-ZOR-Equine was specific to each aspect of the intervention—riding the horse, learning the Zones of Regulation™ curriculum, or engaging with the occupational therapist. Participants primarily found there was a low amount of effort needed to ride the horse, with four children stating it was “easy” or “not that hard” to ride, and Maya affirmed it was a “medium” level of difficulty. For learning Zones of Regulation™ content, there were varied responses. Maya, Joe, and John found it was “easy”; however, John also stated it was “hard” because he felt bored during the Zones of Regulation™ activities. Sam and Ben expressed an intermediate level of effort. Sam indicated learning Zones of Regulation™ was “medium” difficulty, and Ben stated it was “a little tricky”. Engaging with the occupational therapist was “easy” or “not that hard” for four participants, and Maya was unsure of how difficult it was for her to interact with the occupational therapist. Additionally, John expressed concerns with the logistical feasibility of OT-ZOR-Equine. He highlighted the burden of TGEC being “far away” and his discomfort during the hot car ride to travel to the riding center during the summer. John also mentioned his dislike of the saliva collection process used to collect data for the ongoing RCT.

### 3.3. Perceived Effect of the Intervention on Participants’ Emotional State and Self-Regulation

Table 5 provides selected participant responses that illustrate participants’ perceptions on how the intervention did or did not influence their self-regulation. While riding the horse, the children predominately reported feeling positive emotions. Five participants indicated they felt “calm”, four were “happy” or “excited”, and two felt “good” while riding (See Table 5). Maya stated she felt “comfortable” riding the horse. None of the six children mentioned that they felt mad, and five children never felt “uncomfortable” while on the horse. Furthermore, five participants confirmed that riding influenced their emotional management. The children mentioned that riding changed their emotional state, and they felt calmer or more focused after horseback riding. Four children indicated that riding helped them feel better when they were feeling uncomfortable or mad. For example, John noticed he felt happier after riding the horse. Ben explained that the speed of the horse influenced how he felt; when the horse trotted, he felt excited, but when the horse walked slowly, he felt calm. However, four participants also experienced negative emotions while riding. John felt “dirty” and “funny” when touching the horse’s hair and initially “scared” to fall off the horse. Ben felt “nervous” after his fall from the horse, and Sam felt “surprised” when the horse trotted. Joe felt “uncomfortable” riding backward and “bored” while riding. Additionally, only Joe indicated that riding had no noticeable effects on his self-regulation; he did not think that riding helped him feel better or calmer when he was uncomfortable.

For the Zones of Regulation™ aspects of the intervention, participants also experienced positive feelings overall. Four participants indicated that they felt “calm”, two were “excited” or “happy”, and Ben felt “good” while learning the Zones of Regulation™ curriculum. Three children stated that they did not feel mad, and two children never felt uncomfortable engaging in the Zones of Regulation™ curriculum. Additionally, four participants reported that learning the Zones of Regulation™ curriculum had a positive influence on their ability to manage their emotions or change their emotional state. They explained that the Zones of Regulation™ curriculum changed how they manage emotions at home or in the community or helped them to feel calmer. Maya also noted that learning the Zones of Regulation™ helped her to feel more focused and allowed her to help herself feel better. Ben mentioned that he learned that “everything’s not a big deal.” The children emphasized specific self-regulation tools they learned through the Zones of Regulation™ activities that they now use in their daily lives. Maya and Ben stated that they used multiple types of breathing at home or their community recreation center, and Sam affirmed that breathing helped him feel calmer. Sam also thinks about the Zones of Regulation™ curriculum at home, and Joe said he may use the Zones of Regulation™ tools in the future. Hanna mentioned jumping on the trampoline as a tool for self-regulation that she uses at home. However, three participants encountered negative emotions during the Zones of Regulation™ learning activities and one participant did not notice changes in their self-regulation. John and Joe reported feeling “bored”, and Hanna did not feel excited by the Zones of Regulation™ content. John explained that he saw no difference in his self-regulation because he was already familiar with the content.

### 3.4. Participants’ Self-Efficacy in Participating in the Intervention

Table 6 provides selected participant responses that illustrate participants’ self-efficacy in participating in OT-ZOR-Equine. The children’s self-efficacy, or confidence, in participating in OT-ZOR-Equine varied, but was higher for riding the horse compared to learning the Zones of Regulation™ curriculum. Maya, Sam, and Joe felt confident about riding (see Table 6). In contrast, John mentioned that during the first session, he was not confident about staying on the horse. However, the more time he spent riding the horse, his confidence slightly improved, but he was still uncertain. Ben chose to skip the question, and Hanna’s answer was unclear. Only three participants shared their self-efficacy regarding learning the Zones of Regulation™ curriculum. Maya and Sam were confident in learning the Zones of Regulation™ curriculum, and Joe was unsure of his confidence level.

## 4. Discussion

We aimed to describe the aspects of OT-ZOR-Equine that were acceptable or unacceptable to participating autistic children. Based on the children’s attitudes toward the intervention, their perceived burden and self-efficacy in participating, and their perceptions of the effect of the intervention on their self-regulation, we conclude that the children generally found OT-ZOR-Equine to be an acceptable intervention. This is also supported by the fact that all children expressed they would want to return to the Temple Grandin Equine Center to ride horses again. The children especially enjoyed riding and interacting with the horse. However, some participants also considered aspects of the intervention to be less acceptable. Multiple children indicated that they did not enjoy the Zones of Regulation™ curriculum, although two out of those three participants did find the Zones of Regulation^TM^ curriculum helpful in improving their self-regulation skills. In the following paragraphs, we will discuss the less-preferred aspects of the intervention, suggestions for improving acceptability of self-regulation interventions, and how this study compares with other investigations of autistic children’s acceptability of healthcare interventions.

### 4.1. Perceived Difficulty of Self-Regulation-Focused Interventions

It is evident from the data that the children greatly enjoyed engaging with and riding the horse, but they were not as satisfied with the Zones of Regulation™ curriculum. Although half of the participants said it was “easy” to participate in the Zones of Regulation™ learning activities, and multiple participants experienced positive feelings, most children did not want to continue learning about the curriculum. The curriculum focused on understanding emotions and self-regulation, both of which are often difficult for autistic children (Kuypers, 2011; Mazefsky & White, 2013; Rieffe et al., 2006; Samson et al., 2014, 2015). Therefore, it is not surprising that a sample of children with impaired self-regulation and emotional awareness found that working on these topics during the Zones of Regulation™ activities was less preferred. Despite the challenges, self-regulation skill development is still a worthy pursuit, given that self-regulation is consistently identified as an outcome that matters to autistic people (Autistic Self Advocacy Network, 2022), and poor self-regulation skills can have detrimental effects on life outcomes (Nader-Grosbois & Mazzone, 2014; Konstantareas & Stewart, 2006; Swain et al., 2015). Participants in this study used breathing strategies and a variety of other individualized self-regulation tools that they learned during the intervention to support their self-regulation at home and in the community. The children also noticed that they felt calmer or more focused from learning the Zones of Regulation™ curriculum. These perceived improvements in self-regulation align with previous research that occupational therapy using Zones of Regulation concepts can improve irritability, hyperactivity, and emotional dysregulation in autistic children (Peters et al., 2024a). Similarly, other self-regulation interventions for autistic children have been found to improve self-regulation skills, adaptive behaviors, and anger, and decrease the length and number of emotional outbursts (Reyes et al., 2019; Scarpa & Reyes, 2011; Sofronoff et al., 2006; Weiss et al., 2018). Although potentially difficult for autistic children, engaging in self-regulation-focused interventions can lead to better outcomes and quality of life (Reyes et al., 2019; Scarpa & Reyes, 2011; Sofronoff et al., 2006; Weiss et al., 2018). Therefore, it is important to find ways to develop self-regulation interventions that are more acceptable and enjoyable to autistic children.

### 4.2. Suggestions to Improve Acceptability of Self-Regulation Interventions

Integrating a horse into therapeutic activities may be one such tool to improve the acceptability of self-regulation interventions, as the participants of this study described a primarily positive attitude toward and interest in riding horses. They cited many preferred elements of riding, including trotting, playing mounted games, the sensory experience of riding the horse, and the horse’s calmness. They also found that it was an easy or medium level of difficulty to ride the horse. Best practices for working with autistic children include integrating autistic children’s strengths and interests into interventions (Tomchek & Koenig, 2016). Research has also found that including their interests can increase engagement in an intervention (Baker et al., 1998) and improve social interaction (Corbett et al., 2014). Llambias et al. (2016) found that involving a horse during an occupational therapy intervention increased engagement in autistic children. Thus, integrating horses into self-regulation interventions may be an acceptable option to some autistic children to increase engagement and enjoyability of the intervention. Future research should focus on how to best integrate horses into therapeutic interventions to support autistic children’s participation.

The children proposed diverse suggestions to improve the acceptability of OT-ZOR-Equine, including having comfort items on the horse, increased speeds of the horse, or shorter, less messy riding sessions. One participant preferred riding backward, while it made another participant feel uncomfortable. These unique preferences and non-preferred factors expressed by the children further underscore the importance of maintaining client-centeredness in occupational therapy. Occupational therapy practitioners implement client-centered practices by respecting and including clients’ choices and values throughout the therapy process (Law et al., 1995; Sumsion, 2000). A review by Law et al. (1995) found that client-centered care across multiple disciplines resulted in increased client satisfaction and compliance with healthcare interventions. Additionally, in a critical review of client-centered care, Whalley Hammell (2013) emphasized the importance of the intentional implementation of client-centered care by occupational therapists within systems that often emphasize standardization, to ensure that clients are satisfied and engaged in the therapy process. Therefore, to increase the acceptability of OT-ZOR-Equine, we recommend that occupational therapists make client-centered modifications as needed to increase each participant’s engagement and enjoyment.

Additionally, many of the children’s varied preferences, dislikes, or emotions they experienced during the intervention related to increased sensory input (e.g., trotting, feeling the saddle or dirt, etc.). This highlights the sensory differences often present in autistic individuals, including both heightened and diminished responsiveness to sensory input (APA, 2013). It also further emphasizes the need for modifying interventions to meet each client’s sensory needs and preferences.

Participants appreciated their relationship with the occupational therapists throughout the intervention, even though the occupational therapists guided participants through less-preferred aspects of OT-ZOR-Equine, such as the Zones of Regulation™ curriculum. Although some participants felt negative emotions (i.e., nervous, uncomfortable, dirty, bored) at times, they mentioned the kindness and safety that they felt in the occupational therapists’ presence. This highlights the impact that therapeutic relationships between a client and occupational therapist can have on a client’s experience. This was confirmed in a meta-ethnography by Gagne-Trudel et al. (2023) that found therapeutic relationships between children, caregivers, and occupational therapists supported participation in therapy and occupational performance results. This suggests that therapeutic relationships play a role in intervention acceptability to participants.

Some participants in this study had difficulty understanding more abstract concepts, such as confidence, during interviews. There were fewer responses given to questions related to confidence compared to other topics. This may reflect autistic individuals’ challenges with communication, specifically with understanding speech and use of “overly literal language” (APA, 2013, p. 53). According to Gabriels and Hill (2007), it is considered best practice to avoid using abstract concepts when communicating with autistic children. Future acceptability studies should aim to utilize concepts that are more concrete or further modify the language to be more appropriate for this population.

### 4.3. Comparison to Acceptability of Other Healthcare Interventions

The children experienced many positive emotions while riding. This aligns with a review of animal-assisted interventions that found including animals in interventions increased positive emotions in autistic individuals (O’Haire, 2017) and a meta-analysis of equine-assisted services that found across several studies that participating autistic youth had decreased irritability (Xiao et al., 2023). In the current study, participants also noticed a difference in their self-regulation, noting that riding helped to improve their emotional state when they were uncomfortable or upset. They felt calmer, more focused, or happier after riding the horse. Similar findings occurred in the study of Tan and Simmonds (2018) in which caregivers reported improved self-regulation in their autistic child after engaging in equine-assisted services. Gabriels et al. (2015) also found that autistic youth who participated in adaptive horseback riding experienced decreased irritability and hyperactivity. Therefore, in the current study, children perceived OT-ZOR-Equine to be effective in improving various aspects of self-regulation, which is consistent with the extant literature focused on the efficacy of equine-assisted services.

The general acceptability of the OT-ZOR-Equine intervention is consistent with previous studies on OT-ZOR-Equine and occupational therapy in equine environments in which caregivers of autistic youth and the providing occupational therapists found occupational therapy interventions that integrated horses to be acceptable (Peters et al., 2021, 2024b; Kalmbach et al., 2020). This study’s data collection methods were also similar to another autism acceptability study on a virtual nutrition intervention for autistic adolescents. Buro et al. (2023) used focus groups and interviews to understand the participants’ and caregivers’ attitudes towards the intervention. In contrast, an acceptability and feasibility study on the Emotional Awareness and Skills Enhancement (EASE) program for autistic adolescents used the “treatment satisfaction scale” in which adolescents rated their perspectives of the intervention using a ten-item, five-point scale (Conner et al., 2019, p. 1280). However, acceptability studies that include the perspectives of autistic children who participate in the intervention seem to be rare, as most of the acceptability studies that we found for this population only included the perspectives of their caregivers or intervention providers (Peters et al., 2021, 2024b; Kalmbach et al., 2020; Garcia et al., 2024; Shaffer et al., 2019). It is vital to prioritize the perspectives of autistic children who receive therapeutic interventions to ensure the acceptability of the interventions to this population (Autistic Self Advocacy Network, 2022). Therefore, future autism research needs to include the voices of this population throughout the development and evaluation of all interventions that autistic children engage in.

### 4.4. Limitations

This study is limited by the reliance on verbal interviews that asked participants to recall past events, particularly given the difficulty with verbal communication that many autistic children experience. Some participants had challenges remembering aspects of the intervention and may have benefited from images portraying the intervention activities in which they engaged. Additionally, we included questions that were less concrete in the interview, as some of the children were able to understand and answer them. However, not all participants may have understood the more abstract questions (e.g., “How confident did you feel about riding your horse?”), which may have impacted the accuracy of their responses. Further, it is possible that children were providing answers that they perceived the interviewer wanted to hear, which may have impacted the accuracy of responses. To protect against this, we selected an interviewer (JB) who was not involved in intervention delivery, and she began each interview by letting participants know there were no right or wrong answers. Notwithstanding, the possibility remains that children expressed more favorable views of the intervention because they perceived it as the socially desirable response. For all of these reasons, we recommend future research include other methods of assessing acceptability that do not only rely on verbal recall, such as use of an in-the-moment affect rating scale during the intervention.

Additionally, the combination of the inherent social communication challenges of the autistic individuals in this study and the use of an interviewer who was novel to the participants may have also impacted the participants’ ability to answer questions. The use of a familiar interviewer in future research may support the participants’ comfortability and ability to share their perspective. We also did not look at all aspects of The Framework of Acceptability by Sekhon et al. (2017, 2022) in this study, as we believed some concepts from the framework would be too abstract for participants to understand. Future research could address the ethicality, intervention coherence, and opportunity costs (Sekhon et al., 2022) of OT-ZOR-Equine or similar interventions.

Finally, this study was also limited by its small sample size of children recruited from only one cohort of the larger RCT. While *n* = 6 is consistent with other interview-based studies of autism intervention acceptability from a parent (Im-Bolter & de la Roche, 2023) or provider perspective (Chrétien-Vincent et al., 2023), it is not without limitations. Children in different cohorts may have had different opinions of the intervention. Similarly, most children in this study came from relatively higher income households, likely a reflection of the requirement that a parent or caregiver be available to attend the intervention with them (thus biasing the sample against single working parent households or families with two working parents). Future research should examine acceptability of OT-ZOR Equine and other interventions for autistic children in a larger, more diverse sample.

## 5. Conclusions

OT-ZOR-Equine was generally acceptable to autistic children in this study. Most children expressed enjoyment and confidence in riding horses during OT-ZOR Equine and believed that riding helped them feel calm. Acceptability towards learning about self-regulation skills was more varied, with mixed attitudes towards the curriculum and their confidence engaging in it, despite most children endorsing its effectiveness in helping them to manage their emotions. Findings from this study underscore the importance of client-centered care in occupational therapy, particularly adapting standardized interventions to maximize autistic children’s enjoyment and engagement. Horses may be one such tool for increasing autistic children’s engagement in potentially difficult interventions. Overall, findings support the continued use and research of OT-ZOR-Equine to address self-regulation in autistic children, with individualized adaptations to maximize participant acceptability of the intervention.

## Figures and Tables

**Table 1:** Participant characteristics.

Pseudonym	Sex	Age	Race/Ethnicity	ABAS-3 Composite	SCQ	Non-Verbal IQ	Diagnoses	Household Income
Joe	Male	7	White/Hispanic	80	17	110	Autism, Asthma	$81,000–$100,000
Sam	Male	8	Multi/Not Hispanic	87	13	105	Autism, ADHD, Developmental Delays	>$160,000
Hanna	Female	7	Asian/Not Hispanic	84	24	93	Autism	$51,000–$65,000
Ben	Male	8	White/Not Hispanic	75	16	108	Autism, ADHD	>$160,000
Maya	Female	7	White/Not Hispanic	68	13	93	Autism	>$160,000
John	Male	9	Asian/Not Hispanic	75	27	113	Autism	>$160,000

ABAS-3 = Adaptive Behavior Assessment System, Third Edition; SCQ = Social Communication Questionnaire.

**Table 2:** Semi-structured interview guide.

Attitude Towards the Intervention	
	Did you like riding your horse? If yes, what did you like about riding your horse? What is your favorite part about riding your horse? If no, what did you not like about riding your horse? Did you like learning about zones/feelings with your horse? If yes, what did you like about learning zones/feelings with your horse? What is your favorite part about learning zones/feelings with your horse? If no, what did you not like about learning zones/feelings with your horse? Did you like being with [their therapist’s name]? If yes, what did you like about being with [their therapist’s name]? What is your favorite part about being with [their therapist’s name]? If no, what did you not like about being with [their therapist’s name]?
Burden to Participate	
	4. How hard was it to ride your horse? 5. How hard was it to learn about the zones/feelings with your horse? 6. How hard was it to be with [their therapist’s name]?
Perceived Effectiveness on Self-regulation	
	7. How did you feel when you were riding your horse? Did you feel calmer when you were riding your horse? Did you feel excited when you were riding your horse? Did you feel mad when you were riding your horse? Did you feel uncomfortable when you were riding your horse? 8. Do you think riding your horse has made it easier for you to feel calm or focused? If yes, can you tell me about how you think riding your horse has helped you feel calmer? If no, can you tell me more about that? 9. Do you think riding your horse helps you feel better when you are mad or uncomfortable? If yes, can you tell me about how you think riding your horse has helped you feel better? If no, can you tell me more about that? 10. How did you feel when you were learning about zones/feelings? Did you feel calmer when you were learning about zones/feelings? Did you feel excited when you were learning about zones/feelings? Did you feel mad when you were learning about zones/feelings? Did you feel uncomfortable when you were learning about zones/feelings? 11. Do you think learning about zones/feelings has made it easier for you to feel calm or focused? If yes, can you tell me about how you think learning about zones/feelings has helped you feel calmer? If no, can you tell me more about that? 12. Do you think learning about zones/feelings helps you feel better when you are mad or uncomfortable? If yes, can you tell me about how you think learning about zones/feelings with your horse has helped you feel better when you are mad or uncomfortable? If no, can you tell me more about that? 13. Did learning about zones/feelings change how you regulate/manage your emotions at home or school? If yes, can you tell me more about how learning about zones/feelings with your horse changed how you regulate/manage your emotions at home or school? If no, can you tell me more about that? 14. Are there any tools/strategies you have learned that help you feel better when you are at home or school?
Self-Efficacy in Participation	
	15. How confident did you feel about riding your horse? 16. How confident did you feel about learning about zones/feelings with your horse?
Suggested Improvements	
	17. What did you not like about riding your horse? 18. What would make riding your horse more comfortable or fun? 19. What did you not like about learning about zones/feelings with your horse? 20 What would make learning about zones/feelings with your horse more comfortable or fun?
General Acceptability	
	21. Would you want to go back to Temple Grandin Equine Center to ride your horse again? 22. Would you want to go back to Temple Grandin Equine Center to learn about zones/feelings with your horse again?

**Table 3:** Attitudes toward the intervention. Selected participant responses that best illustrate the theme.

Subcodes	Participant Quotes
Liked Being with the Horse	Ben: I don’t think there was anything I didn’t like riding on Bluebell. John: I like riding horses overall. Joe: I would even pay $50,000 to buy Concho. I want Concho as my pet.
Non-preferred Factors of Riding	Ben: And there was another day, um the day after I fell off where I was nervous because she was twitching a lot and I felt that was like, and that kind of made me nervous. John: I already said that he’s too messy Joe: Only when I had to ride in the saddle backward…It’s the it’s the most uncomfortable thing in the entire world.
Liked Being with Occupational Therapist	Ben: That she would always, all the time, she would tell me the instructions um when I was doing something John: Um. She’s really nice. John: She always makes sure that I’m really safe.
Liked Learning Zones of Regulation™ Curriculum	Interviewer: Did you like learning about feelings with your horse? Maya: Mm-hmm. Interviewer: Do you want to come back to the Temple Grandin Center to learn about feelings? Sam: Yeah
Did Not Like Learning Zones of Regulation™ Curriculum	John: It was the most boring thing…. because I already know a lot about zones… I felt like like why are you even doing this? I already know all of this. Interviewer: Did you like learning about zones with Concho? Joe: I hate it… it was boring. Interviewer: Would you want to come back here to learn about feelings with Harley? Maya: Hmm nah.
Suggestions for Riding Horse	Interviewer: What would make riding your horse more fun? Hanna: If if, if llama [participant’s stuffed animal] was there…If we eat s’mores John: I wish the time I was on Chunky was shorter. Not because I don’t like it, but because it’s just really long Interviewer: What would make riding Flyer more fun? Sam: Trotting.
No Suggestions for Riding Horse	Interviewer: Was there anything you didn’t like about riding Concho? Joe: No Interviewer: What would make riding Concho more comfortable or more fun? Joe: Nothing. It’s good as it is.
Suggestions for the Zones of Regulation™ Curriculum	Interviewer: What would make learning about feelings more comfortable or more fun? Hanna: Llama [participant’s stuffed animal]. Interviewer: Having your llama with you? Interviewer: What would have made it more fun to learn about zones? Joe: If I got to gallop.
No Suggestions for the Zones of Regulation™ Curriculum	Interviewer: What would make learning about feelings more fun? Sam: Uhh. I don’t know.

**Table 4:** Burden to participate in the intervention. Selected participant responses that best illustrate the theme.

Subcodes	Participant Quotes
Easy to Ride Horse	Interviewer: How hard was it to ride Concho? Joe: Easy…Easy peasy lemon squeezy
Medium to Ride Horse	Interviewer: Was it easy, medium, or hard to ride Harley? Maya: I think medium.
Easy to Learn the Zones of Regulation™ Curriculum	Interviewer: How hard was it to learn about zones? Joe: Easy. Easy. Easy.
Medium to Learn the Zones of Regulation™ Curriculum	Interviewer: How about learning feelings? Was that easy, medium, or hard? Sam: Medium. Interviewer: How hard was it to learn about zones? Ben: A little tricky
Hard to Learn Zones of Regulation™	Interviewer: How hard was it to learn about zones? John: Hard because like it was boring to boring to learn about again.
Easy to Be with Therapist	Interviewer: And about how hard was it to hang out with Erin? Sam: Hmm. Easy.
Unsure of Difficulty Being with Therapist	Interviewer: How hard was it to be with Mary? Maya: I don’t know
Logistical Feasibility Concerns	John: I just don’t like that it’s so far away and about and like the saliva sample… It was hot when we drive there.

**Table 5:** Perceived effect of the intervention on participants’ emotional state and self-regulation. Selected participant responses that best illustrate the theme.

Subcode: Feelings Experienced While Riding	
Subcodes	Participant Quotes
Calm	Interviewer: How did you feel while you were riding him? John: Relaxed.
Happy	Interviewer: How did you feel while you were riding Harley? Maya: Happy.
Good	Ben: Um Good. Interviewer: You felt good when the horse trotted? Ben: Mm-hmm.
Comfortable	Maya: More comfortable. Interviewer: It was comfortable riding Harley? Maya: Mm-hmm.
Excited	Interviewer: You felt good when the horse trotted? Ben: It it made me feel, like excited.
Surprised	Sam: Ah, surprised Interviewer: What about riding flyer made you feel surprised? Sam: Like the trotting.
Not Uncomfortable	Interviewer: Did you feel uncomfortable while you’re riding your horse? Hanna: No.
Not Mad	Interviewer: Did you feel mad when you were riding your horse? Hanna: No.
Bored	Interviewer: Did you feel calm while you were riding Concho? Joe: Bored.
Uncomfortable	Interviewer: Did you ever feel uncomfortable while you were riding Concho? Joe: Only when I had to ride in the saddle backwards… It’s the it’s the most uncomfortable thing in the entire world.
Nervous	Ben: So there So there was that one day I fell off and there was another day, um the day after I fell off where I was nervous because she was twitching a lot and I felt that was like, and that kind of made me nervous. John: So at first I felt scared because I thought I might fall down because it would really hurt because we’re really up high on a horse and horses are really tall and they’re almost tall and and Chunky’s taller than me.
Dirty	John: Also dirty because Chunky kept doing a thing a thing, and then he kept splashing like that like the dust on us.
Funny	John: I tried to put, I tried putting Chunky’s hair on the other side. Interviewer: Yeah. How did that make you feel? John: Uh funny.
Subcode: Feelings Experienced Learning the Zones of Regulation™ Curriculum	
Subcodes	Participant Quotes
Calm	Interviewer: How else did you feel while you’re learning about feelings? Maya: Just chill.
Good	Interviewer: How did you feel while you were learning about zones? Ben: Um good.
Happy	Interviewer: How did you feel when you were learning about feelings? Hanna: Hmm. Happy.
Excited	Interviewer: Did you feel excited while you were learning about feelings? Sam: Uh yeah.
Not Uncomfortable	Interviewer: Did you feel uncomfortable while you were learning about feelings? Sam: Uh no.
Not Mad	Interviewer: What about did you feel mad while you were learning about feelings? Hanna: No.
Not Excited	Interviewer to Hanna: How about did you feel excited while you were learning about feelings? Interviewer: No? [interviewer’s response to Hanna’s head shaking ‘no’]
Bored	Interviewer: Did you like learning about zones while you were with Chunky? John: It was the most boring thing…because I already know a lot about zones.
Subcode: Riding Affects Self-Regulation	
Subcodes	Participant Quotes
Riding Influences Emotional Management or Emotional State	Interviewer: Do you think riding Harley has made it easier for you to feel calm or focused? Maya: Focused. Interviewer: Do you think riding Flyer has made it easier for you to feel calm or focused? Sam: Yeah. Sam: It makes me feel better. John: I think there’s if I was not feeling good like before I rode chunky. Then I was really happy after.
No Noticeable Effects on Self-Regulation from Riding Horse	Interviewer: Do you think riding Concho helps you feel better when you’re mad or uncomfortable? Joe: No.
Subcode: The Zones of Regulation™ Curriculum Affect Self-Regulation	
Subcodes	Participant Quotes
The Zones of Regulation™ Curriculum Influences Emotional Management or Emotional State	Interviewer: What about… do you think learning about feelings with Harley has changed how you manage your emotions at home or at school? Maya: Umm. At home. Interviewer: Do you think learning about feelings has made it easier for you to feel calm? Sam: Yeah. Interviewer: Is there anything else you learned while you were with Bluebell that you use at the Y? Ben: Well, I, I don’t, maybe, um, everything’s not a big deal.
Tools from the Zones of Regulation™ Curriculum Used in Daily Life	Interviewer: Is there anything you learned while you’re with Harley that you do while you’re at home? Maya: Breathing… I mean…the lazy eight…And the six eight breathing right… Palm press. Interviewer: What kind of things did you learn that you use at home? Sam’s Father: Do you think about the zones? Sam’s Father: Sometimes you think about the zones and how you’re feeling? Sam: Yeah. Interviewer: Did you learn any tools that help you at other places? Ben’s Mother: At the YMCA? Ben: Uh-huh. Interviewer: Hmm what kind of tools did you learn? Ben: Um lazy eight and belly breath.
No Noticeable Effects on Self-Regulation from the Zones of Regulation™ Curriculum	Interviewer: Ok so do you think learning about zones has made it easier for you to feel calm? John: I mean I already knew about zones in the first place so it didn’t really help because I already knew about zones. I learned at school. Interviewer to Hanna: Do you think learning about feelings with your horse helps you feel better when you’re mad or uncomfortable? Interviewer: No? [Interviewer’s response to Hanna’s head shaking ‘no’].

**Table 6:** Self-efficacy in participating in the intervention. Selected participant responses that best illustrate the theme.

Subcode	Participant Quotes
Confident Riding the Horse	Interviewer: Did you feel confident while you were riding Flyer? Sam: Uh yeah.
Not Confident Riding the Horse	Interviewer: How confident did you feel riding Chunky? John: So at first I felt scared because I thought I might fall down.
Unsure of Confidence Riding the Horse	Interviewer: How did you feel after riding him for a little bit? John: I felt more safe, but still like, not perfect.
Confident Learning the Zones of Regulation™ Curriculum	Interviewer: Did you feel confident while you were learning about feelings? Joe: Uh yeah.
Unsure of Confidence Learning the Zones of Regulation™ Curriculum	Interviewer: How confident did you feel learning about zones with Concho? Joe: I don’t know.

## Data Availability

The raw data supporting the conclusions of this article will be made available by the authors on request.
